# HAVIT: research on vision-language gesture interaction mechanism for smart furniture

**DOI:** 10.1038/s41598-025-10758-9

**Published:** 2025-07-28

**Authors:** Hong Chen, Hasnul Azwan Azizan Mahdzir, Xuekun Li, Nurul Ayn Ahmad Sayuti

**Affiliations:** 1 School of Art and Design, Jiangxi University of Technology, Nanchang, China; 2 Faculty of Art & Design, Universiti Teknologi MARA, Shah Alam, Malaysia; 3 College of Art, Guizhou University of Engineering Science, Bijie, China

**Keywords:** Biological techniques, Biotechnology, Computational biology and bioinformatics, Neuroscience, Physiology

## Abstract

With the rapid development of smart furniture, gesture recognition has gained increasing attention as a natural and intuitive interaction method. However, in practical applications, issues such as limited data resources and insufficient semantic understanding have significantly constrained the effectiveness of gesture recognition technology. To address these challenges, this study proposes HAVIT, a hybrid deep learning model based on Vision Transformer and ALBEF, aimed at enhancing the performance of gesture recognition systems under data-scarce conditions. The model achieves efficient feature extraction and accurate recognition of gesture characteristics through the organic integration of Vision Transformer’s feature extraction capabilities and ALBEF’s semantic understanding mechanism. Experimental results demonstrate that on a fully labeled dataset, the HAVIT model achieved a classification accuracy of 91.83% and an AUC value of 0.92; under 20% label deficiency conditions, the model maintained an accuracy of 86.89% and an AUC value of 0.88, exhibiting strong robustness. The research findings provide new solutions for the development of smart furniture interaction technology and hold significant implications for advancing practical applications in this field.

## Introduction

### Background

With the rapid development of emerging information technologies such as artificial intelligence and Internet of Things, intelligence and digitalization are profoundly transforming human lifestyles and production modes. In this wave of technological revolution, furniture, as the most direct carrier of human daily life, is undergoing a critical transition from traditional functions to intelligent upgrades. According to Grand View Research^1^, the global smart furniture market reached $143.6 million in 2020 and is projected to expand at a compound annual growth rate of 12.4% during 2021-2028. This growth trend aligns with the robust development of the global smart home market—Business Insider^2^ reports that the smart home market is expected to grow from $78.3 billion in 2020 to $135.3 billion by 2025. The statistical platform Statista^3^ predicts that in the home office furniture sector, the global market size will reach $49.6 billion by 2024.

**Fig. 1:**
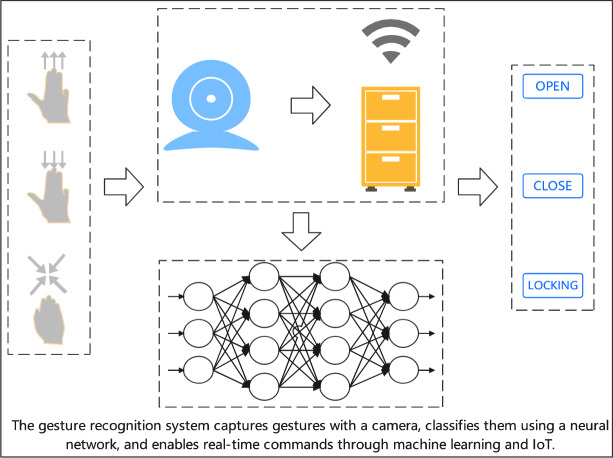
Gesture Recognition for Smart Furniture.

The development of smart furniture not only relates to the transformation and upgrading of the furniture manufacturing industry but is also closely tied to improving human quality of life, as shown in Fig. 1. However, smart furniture development currently faces a key challenge in interaction methods. Existing human-computer interaction technologies struggle to adapt to diverse usage scenarios, showing obvious limitations in convenience and intuitiveness. Particularly in complex and varied home environments, traditional control methods such as remote controllers and mobile applications can no longer meet user demands.

Gesture recognition, as a cutting-edge technology in human-computer interaction, demonstrates significant application potential due to its natural and intuitive characteristics, supported by computer vision and deep learning technologies. The post-pandemic era’s demand for contactless interaction further highlights the practical value of this technology in scenarios such as smart furniture. However, in actual application environments, improving the accuracy and environmental adaptability of gesture recognition systems still faces significant challenges due to factors such as limited data resources and scene complexity. Through innovative applications of deep learning methods, this research aims to address these technical challenges and provide new theoretical foundations and solutions for the practical application of intelligent interaction technology.

### Literature review

The development of gesture recognition technology has opened new possibilities for smart furniture interaction. Through a systematic analysis of gesture recognition research over 25 years, Alyami et al. revealed the evolution trend from traditional methods to deep learning but pointed out that existing research overly focuses on sign language recognition, with insufficient application research in universal interaction scenarios^1^. Han et al. proposed a method combining grey wolf optimization algorithm and 3D reconstruction, enhancing recognition effects through the fusion of geometric features, 3D point modeling, and corner features, but this method involves complex calculations in feature extraction and fails to consider environmental interference issues in practical scenarios^2^. Ma et al. designed a multimodal network based on motion energy that improved recognition performance, but their method heavily relies on Kinect camera depth information, increasing deployment costs and lacking gesture semantic understanding^3^.

In terms of interaction scenario adaptability research, Zhou et al. proposed the DigCode encoding method, converting continuous actions into discrete string encodings to improve the standardization of gesture expression, but failed to solve the problem of insufficient data volume and ignored the personalized needs of different users^4^. Ji et al. conducted a review of flexible strain sensor gloves, which showed advantages in cost and portability, but the contact-based solution limited user experience^5^. Wang et al.’s systematic study of elderly users found that gesture simplicity and directionality are key factors, but the research was limited to specific user groups and lacked universal verification^6^.

In technical solution exploration, Yu et al.^7^ proposed a dynamic gesture recognition approach based on 2D convolutional neural networks and feature fusion, achieving improved recognition accuracy, but their method still faces challenges in real-time applications with varying lighting conditions typical in smart furniture contexts. Marzouk et al. proposed the ASODCAE-SLR model, combining atomic search optimization and deep convolutional autoencoders, but the model requires large amounts of training data and has limited feature extraction capabilities^8^. Liu et al. achieved high-accuracy gesture recognition using the YOLOv5 algorithm, but it only applies to predefined simple gesture sets and lacks the ability to understand complex interaction intentions^9^. Zhou et al. studied user-defined gestures for multi-scale GIS interfaces, finding that gesture features vary with spatial scale, but did not deeply explore semantic-level adaptability issues^10^.

Recent advances in attention-based models have shown promising results in gesture recognition. Dey et al.^11^ developed an attention-driven C3D-BiLSTM network for recognizing question sign gestures in video streams, demonstrating the effectiveness of attention mechanisms in capturing temporal dynamics of gestures. However, their approach primarily focuses on specific sign language gestures rather than general interaction gestures needed for smart furniture control. Joksimoski et al.’s review emphasized the importance of data collection, feature extraction, and recognition technologies but pointed out that current research still shows insufficient robustness in complex environments^12^. Jeong et al. explored non-verbal sound interaction based on tapping and touching, achieving good recognition results, but this single-modal solution struggles to meet the diverse interaction needs of smart furniture^13^.

To enhance the practicality of smart furniture gesture interaction, gesture recognition research mainly faces two key challenges: data scarcity and semantic understanding, indicating the importance of exploring more effective feature extraction and semantic understanding methods. Table 1’s systematic analysis of existing research further confirms the necessity of this research approach.

**Table 1:** Analysis of related research methods.

Authors	Application scenario	Research content	Potential limitations
Wang et al.^14^	Customizable gesture interface	Lightweight multilayer perceptron architecture based on contrastive learning	Lacks deep understanding of gesture semantics; Strong data dependency
Sharma et al.^15^	Dynamic sign language translation system	Dynamic gesture recognition based on temporal distributed LSTM	High dependency on continuous action sequences; Incomplete gesture feature extraction; Poor recognition in limited samples
Li et al.^16^	Social robot tactile recognition	Decomposed spatiotemporal convolution for feature representation	Insufficient environmental adaptability; Lacks fusion with other perception modalities
Jung et al.^17^	Gesture collection in crowdsourcing	Gesture data collection and recognition through crowdsourcing	Unstable data quality; High annotation cost; Lacks effective solutions for small-scale data
Caraka et al.^18^	Indonesian sign language translation	LSTM gesture recognition based on skeletal points	Lacks semantic understanding; Insufficient environmental robustness
Marzouk et al.^8^	Arabic sign language communication	Optimized deep convolutional autoencoder recognition framework	Insufficient feature extraction; Limited semantic understanding; Unstable performance in small sample scenarios
Chen et al.^19^	Smart bathroom control system	Gesture interaction system without visual feedback	Single interaction mode; Insufficient gesture understanding
Parra-Dominguez et al.^20^	Hand recognition for facial paralysis patients	Hybrid feature gesture recognition method	Limited scene adaptability; Lacks adaptive learning mechanism
Yu et al.^7^	Dynamic gesture control	2D CNN-based gesture recognition with feature fusion	Environmental sensitivity; Limited semantic understanding; Requires significant preprocessing
Dey et al.^11^	Question sign gesture recognition	Attention-driven C3D-BiLSTM network	Specific to sign language; Computational complexity; Limited application to general interaction contexts
Brozdowski et al.^21^	Sign language transition recognition	Research on gesture action continuity	Ignores static features; Lacks overall semantic understanding; Incomplete feature extraction
Stewart et al.^22^	Child pre-language gesture development	Analysis of gesture expression and understanding correlation	Overly specific scenarios; Insufficient method generalization

Analysis of existing research reveals that although these methods have made certain progress in their respective fields, they still show significant limitations in practical application scenarios for smart furniture. Existing research generally requires substantial annotated data support. These issues severely constrain the practical application of gesture recognition technology in the smart furniture domain. Based on this, this research proposes a technical solution combining Vision Transformer (ViT) with Aligned Language-Image BERT for Enhanced Vision-language Understanding (ALBEF), aiming to provide a solution that can simultaneously address the challenges of data scarcity and semantic understanding. The Vision Transformer architecture offers powerful feature extraction capabilities through self-attention mechanisms that capture long-range dependencies in visual data, while ALBEF provides advantages in cross-modal alignment and contrastive learning, enabling better semantic understanding of gestures with limited annotated samples.

### Our contributions

- Addressing the problem of excessive reliance on complete annotated data in existing gesture recognition research, we propose the HAVIT hybrid deep learning model. This model achieves 86.89% accuracy with only 80% annotated data, significantly outperforming existing methods and providing a viable solution for smart furniture interaction in data-resource-limited scenarios.
- We design a collaborative mechanism based on Vision Transformer feature extraction and ALBEF semantic understanding. Through systematic ablation experiments, this mechanism achieves an Area Under the Curve (AUC) value of 0.92, showing significant improvement compared to using ALBEF (0.71) or Vision Transformer (0.76) alone, confirming the effectiveness of this design in enhancing gesture semantic understanding. This innovative integration leverages the complementary strengths of both architectures: Vision Transformer’s ability to capture global spatial relationships and ALBEF’s cross-modal alignment capabilities.
- For complex and varied interaction environments in smart furniture scenarios, we propose a hybrid feature representation method combining visual features and semantic information. Experiments show that this method can achieve high true positive rates in low false positive rate regions, providing a new technical approach for improving the recognition accuracy of smart furniture interaction systems. This hybrid approach addresses a key limitation in existing methods that typically focus on either visual features or semantic context, but rarely both simultaneously.

## Methods

### Problem description

The three-dimensional space where smart furniture is located is defined as $\Omega \in {\mathbb {R}}^3$. In this space, users interact with smart furniture through gestures, and the system needs to accurately map the captured gesture sequences to control commands to achieve natural human-computer interaction. At time *t*, the gesture image sequence captured by the system is represented as:

1 $$\begin{aligned} I_t = \{i_1, i_2,\ldots , i_n\} \in {\mathbb {R}}^{H \times W \times C} \end{aligned}$$

where each image $i_k \in {\mathbb {R}}^{H \times W \times C}$ is a three-dimensional tensor, *H* and *W* represent the height and width of the image respectively, and *C* is the number of image channels. Each frame contains rich spatial information and visual features of gestures. To precisely characterize the position and posture information of gestures in three-dimensional space, we introduce the transformation matrix on the special Euclidean group *SE*(3):

2 $$\begin{aligned} T = \begin{bmatrix} R & p \\ 0 & 1 \end{bmatrix} \in SE(3) \end{aligned}$$

where the rotation matrix $R \in SO(3)$ describes the spatial orientation of the gesture, *SO*(3) represents the three-dimensional special orthogonal group, and the translation vector $p \in {\mathbb {R}}^3$ characterizes the spatial position of the gesture. This representation method can completely describe the six-degree-of-freedom motion state of gestures in three-dimensional space. In the smart furniture control system, all executable control commands form a finite set:

3 $$\begin{aligned} {\mathcal {C}} = \{c_1, c_2,\ldots , c_m\}, \quad c_i \in {\mathcal {A}} \end{aligned}$$

where ${\mathcal {A}}$ is the action space of control commands, and each command $c_i$ corresponds to a specific operation of smart furniture, such as the lifting of smart beds or the switching of smart wardrobes. The core task of the gesture recognition system is to construct a mapping from image space to control command space:

4 $$\begin{aligned} f: {\mathbb {R}}^{H \times W \times C} \rightarrow {\mathcal {C}}, \quad f(I_t) = c_i \end{aligned}$$

Considering the complexity of practical application scenarios, the system also needs to model environmental factors. Define the environment state space:

5 $$\begin{aligned} {\mathcal {E}} = \{e_1, e_2,\ldots , e_k\}, \quad e_i \in {\mathcal {S}} \end{aligned}$$

where ${\mathcal {S}}$ is the set of environmental states, including various factors affecting recognition effectiveness, such as lighting changes $e_1 \in {\mathbb {R}}^+$, occlusion degree $e_2 \in [0,1]$, etc. Based on this, the optimization objective of gesture recognition can be expressed as a conditional probability maximization problem:

6 $$\begin{aligned} \min _{\theta } {\mathcal {L}}(f_{\theta }(I_t|{\mathcal {E}}), y), \quad \theta \in \Theta \end{aligned}$$

where parameter space $\Theta$ contains all possible model parameter configurations, *y* is the true label, and ${\mathcal {L}}$ is the loss function measuring the difference between predicted and true values.

To enhance the robustness and generalization ability of the model, we design an objective function containing regularization terms:

7 $$\begin{aligned} {\mathcal {L}}_{total} = {\mathcal {L}}_{rec} + \lambda {\mathcal {L}}_{reg}, \quad \lambda> 0 \end{aligned}$$

where recognition loss ${\mathcal {L}}_{rec}$ measures the accuracy of model predictions, regularization term ${\mathcal {L}}_{reg}$ controls model complexity, and $\lambda$ is a balance factor weighing the importance of both terms. Based on the above optimization objectives, the system finally outputs the control command with maximum posterior probability:

8 $$\begin{aligned} c^* = \arg max_{c \in {\mathcal {C}}} P(c|I_t, {\mathcal {E}}) \end{aligned}$$

#### Problem 1

In the practical application scenarios of smart furniture, users need to achieve natural interactive control of devices such as smart beds and smart wardrobes through gestures. Considering uncertain factors such as lighting changes and occlusion in the home environment, as well as the practical constraints of difficult annotation data acquisition, this problem can be formally represented as:

9 $$\begin{aligned} \mathop {\arg \min }_{\theta } {\mathbb {E}}_{I_t \sim {\mathcal {D}}} \left[ {\mathcal {L}}_{rec}(f_{\theta }(I_t|{\mathcal {E}}), y) + \lambda {\mathcal {L}}_{reg}(\theta )\right] , \text { s.t. } |{\mathcal {D}}| \ll N \end{aligned}$$

This optimization objective describes how to achieve accurate understanding of user gesture intentions under the constraint of limited annotated data $(|{\mathcal {D}}| \ll N)$. The model needs to cope with various interference factors ${\mathcal {E}}$ brought by the home environment while minimizing the expectation of recognition loss ${\mathcal {L}}_{rec}$ and regularization loss ${\mathcal {L}}_{reg}$ through parameter optimization $\theta$. The solution to this problem directly relates to the practicality of smart furniture interaction systems and has important significance for improving user experience.

### Vision transformer: gesture recognition

#### Comparison with traditional methods: advantages of vision transformer

- Traditional gesture recognition methods mainly rely on Convolutional Neural Network (CNN) architectures, which have obvious limitations: CNN’s local receptive field mechanism struggles to effectively capture long-range dependencies in gesture actions, leading to semantic fragmentation in complex gesture sequence recognition^23,24^. Meanwhile, CNN architectures require large amounts of annotated data to train model parameters, making it difficult to obtain sufficient training samples in emerging application areas such as smart furniture^25^.
- Vision Transformer architecture provides a superior solution through its self-attention mechanism: global attention mechanism can directly model feature correlations at any position in the sequence, effectively solving the long-range dependency problem. Multi-head attention mechanism allows the model to learn features from different representation subspaces, enhancing the ability to recognize subtle differences in gesture actions and improving model performance in complex scenarios.

**Fig. 2:**
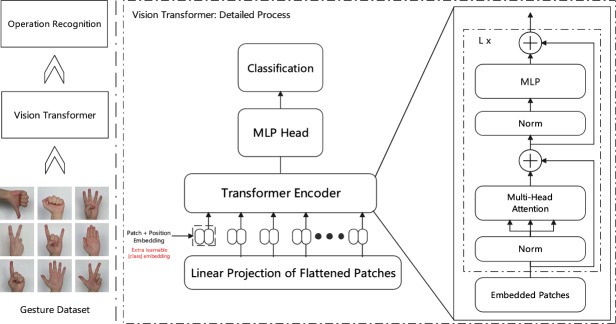
Vision transformer: gesture recognition.

As shown in Fig. 2, the Vision Transformer structure proposed in this research divides the input gesture image into patches and performs linear projection, adding positional encoding information. The processed features are processed through multiple layers of Transformer encoders, each containing multi-head attention mechanism and MLP structure. This design based on global attention breaks through the local receptive field limitations of traditional CNNs, better capturing long-range dependencies between gesture features, and ultimately completing classification prediction through the MLP head.

#### Mechanism of vision transformer in gesture recognition

Vision Transformer (ViT) demonstrates superior performance in gesture recognition tasks by converting images into sequence data and utilizing self-attention mechanisms for feature extraction. To enable Transformer to process two-dimensional image data, the input image $I_t \in {\mathbb {R}}^{H \times W \times C}$ is first divided into fixed-size $P \times P$ patches and transformed through linear mapping and positional encoding to obtain initial embedded representations:

10 $$\begin{aligned} \begin{aligned} z_0 = \left[ x_{class}; \{ \text {Flatten}(I_t^{(i)}) E + E_{pos,i} \}_{i=1}^N \right] \quad \text {where} \quad N = \frac{HW}{P^2}, \quad E \in {\mathbb {R}}^{P^2C \times D}, \quad E_{pos} \in {\mathbb {R}}^{(N+1) \times D} \end{aligned} \end{aligned}$$

where $x_{class} \in {\mathbb {R}}^D$ is the classification token. Next, the multi-layer structure of Transformer processes these embeddings by stacking multiple Transformer blocks. Each Transformer block includes Multi-Head Self-Attention (MSA), Feed-Forward Network (MLP), Layer Normalization, and residual connections. The specific computation process can be expressed as:

11 $$\begin{aligned} \begin{aligned} z'_l&= \text {MSA}(\text {LN}(z_{l-1})) + z_{l-1}, \\ z_l&= \text {MLP}(\text {LN}(z'_l)) + z'_l, \end{aligned} \end{aligned}$$

where the Multi-Head Self-Attention (MSA) mechanism first performs Layer Normalization (LN) on the input features $z_{l-1}$, then applies multi-head self-attention operations, and performs residual connection with input $z_{l-1}$ to obtain intermediate representation $z'_l$. Specifically, the computation process of MSA is:

12 $$\begin{aligned} \text {MSA}(z_{l-1}) = \text {Concat}(\text {head}_1, \dots , \text {head}_H) W^O, \end{aligned}$$

where the computation formula for each attention head $\text {head}_h$ is:

13 $$\begin{aligned} \text {head}_h = \text {softmax}\left( \frac{(z_{l-1} W_Q^h)(z_{l-1} W_K^h)^\top }{\sqrt{d_k}} \right) (z_{l-1} W_V^h). \end{aligned}$$

In the above formula, $W_Q^h, W_K^h, W_V^h \in {\mathbb {R}}^{D \times d_k}$ represent the projection matrices for query, key, and value of the *h*-th attention head respectively, and $W^O \in {\mathbb {R}}^{Hd_v \times D}$ is the projection matrix for multi-head attention output. Layer Normalization operation $\text {LN}$ is used to stabilize the training process, while residual connections help alleviate the gradient vanishing problem. The intermediate representation $z'_l$ obtained through MSA and residual connection is input into the Feed-Forward Network (MLP), and similarly obtains the final output $z_l$ through layer normalization and residual connection. The specific computation process of MLP is:

14 $$\begin{aligned} \text {MLP}(x) = W_2 \sigma (W_1 x + b_1) + b_2, \end{aligned}$$

where $W_1 \in {\mathbb {R}}^{D \times 4D}$ and $W_2 \in {\mathbb {R}}^{4D \times D}$ are the weight matrices for the first and second layers of MLP respectively, $b_1$ and $b_2$ are the corresponding bias terms, and $\sigma$ represents the GELU activation function. Through this structure, MLP can perform nonlinear transformations on features, thereby enhancing the model’s expressive ability. To fully utilize multi-scale features, a feature pyramid attention mechanism is designed to fuse features at different scales through downsampling and attention:

15 $$\begin{aligned} F_{pyr} = \sum _{s=1}^S \text {softmax}\left( W_p \, \text {DownSample}(z_l, s) \right) \cdot \text {Attention}\left( z_l W_Q^s, z_l W_K^s, z_l W_V^s \right) \end{aligned}$$

where $s \in \{1,2,\dots ,S\}$ represents different scale levels, $W_p \in {\mathbb {R}}^{D \times D}$ is the parameter matrix for learning scale weights, and $\text {DownSample}(z_l, s)$ performs level *s* downsampling processing on feature $z_l$. During model training, contrastive learning loss and cross-entropy loss with label smoothing are combined to enhance the model’s generalization ability and discriminative power:

16 $$\begin{aligned} \begin{aligned} {\mathcal {L}}_{total} = \alpha {\mathcal {L}}_{ce} + \beta {\mathcal {L}}_{cont} + \gamma \Vert \theta \Vert _2 \end{aligned} \end{aligned}$$

where ${\mathcal {L}}_{total}$ represents the overall optimization objective, composed of cross-entropy loss with label smoothing ${\mathcal {L}}_{ce}$, contrastive learning loss ${\mathcal {L}}_{cont}$, and L2 regularization term $\Vert \theta \Vert _2$. The complete optimization objective and model inference process can be uniformly expressed as:

17 $$\begin{aligned} \begin{aligned} h&= \text {MLP}(z_L[0])\\ P(c|I_t)&= \text {softmax}(h)\\ c^*&= \arg \max _{c \in {\mathcal {C}}} P(c|I_t) \end{aligned} \end{aligned}$$

where *h* is the final feature representation obtained by processing the classification token feature $z_L[0]$ of the last Transformer layer through multilayer perceptron (MLP). $P(c|I_t)$ represents the predicted probability distribution of class *c* corresponding to input image $I_t$, and $c^*$ is the final gesture class prediction result obtained by taking the maximum value of the predicted probability distribution.

##### Theorem 1

(Multi-scale Feature Representation Theorem) For any gesture image sequence $I_t$ and its feature representation $z_l$ in Vision Transformer, there exists an optimal set of scale weights $\{w_s^*\}_{s=1}^S$, such that the multi-scale feature pyramid representation satisfies:

18 $$\begin{aligned} \Vert {\mathcal {F}}(I_t) - \sum _{s=1}^S w_s^* \cdot \text {Attention}(Q_s, K_s, V_s)\Vert _2 \le \epsilon \end{aligned}$$

where ${\mathcal {F}}(I_t)$ is the ideal feature representation of the image, $\epsilon> 0$ is any small positive number, and $w_s^*$ is determined by the statistical characteristics of the feature distribution. This theorem indicates that through appropriate scale weight combinations, the multi-scale feature pyramid can approximate the ideal gesture feature representation with arbitrary precision.

##### Corollary 1

(Uncertainty Bound of Feature Representation) Based on the Multi-scale Feature Representation Theorem, for any two different gesture samples $I_t^1$ and $I_t^2$, the difference in their feature representations satisfies:

19 $$\begin{aligned} \Vert f(I_t^1) - f(I_t^2)\Vert _2 \ge \delta \Vert I_t^1 - I_t^2\Vert _2 - 2\epsilon \end{aligned}$$

where $f(\cdot )$ represents features extracted through multi-scale Vision Transformer, and $\delta> 0$ is a constant related to the model architecture. This inequality provides theoretical guarantees for the discriminative ability of feature representation.

### ALBEF: interactive intent recognition

#### Comparison with traditional methods: potential of ALBEF in interactive intent recognition

- Traditional gesture recognition methods typically use predefined gesture-command mappings or simple classification models for intent understanding. These approaches lack deep understanding of the potential associations between gesture actions and interaction intents^26,27^. In practical applications, the same gesture may express different interaction intents due to different contextual scenarios, and traditional methods struggle to address this challenge of semantic diversity^28^.
- The ALBEF (Align before Fuse) model can establish semantic mapping relationships between gesture features and interaction intents through cross-modal contrastive learning mechanisms. Its contrastive learning strategy not only learns more discriminative feature representations from limited annotated data but also enhances the model’s ability to understand gesture semantics through contrasting. This contrastive learning-based approach is particularly suitable for interaction intent recognition in smart furniture scenarios, better handling the diversity and ambiguity of gesture semantics.

**Fig. 3:**
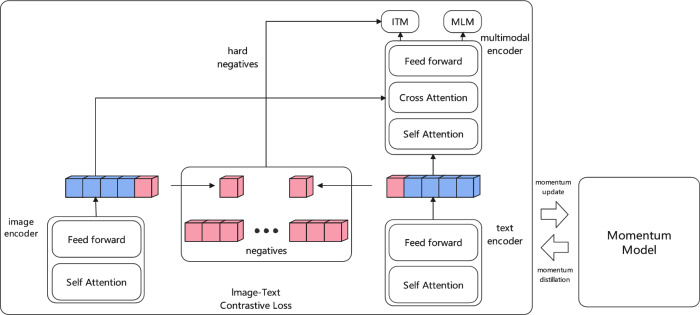
ALBEF: Interactive Intent Recognition.

Figure 3 shows the overall architecture of the ALBEF model, which includes two main branches: an image encoder and a text encoder. Both encoders adopt a combined structure of self-attention mechanism and feed-forward network to extract features from their respective modalities. Through image-text contrastive loss functions, the model learns to establish semantic mapping relationships between gesture image features and interaction intent text. The model also introduces a multimodal encoder that enhances feature fusion through a combination of self-attention, cross-modal attention, and feed-forward networks, ultimately optimizing model parameters through momentum update mechanism to improve gesture semantic understanding capability.

#### Mechanisms in ALBEF model

The ALBEF model receives Vision Transformer’s output as visual feature input and achieves gesture intent understanding through cross-modal alignment and fusion mechanisms. In our approach, we adapt ALBEF specifically for gesture recognition by associating each gesture with corresponding text descriptions that represent its semantic meaning in smart furniture control. This allows the model to establish semantic connections between visual gestures and their intended functions. Let the input image be $I \in {\mathbb {R}}^{H \times W \times 3}$ and the text sequence be *T*. Feature enhancement based on Vision Transformer output:

20 $$\begin{aligned} \begin{aligned} v_{init}&= \{z_L^{(i)}\}_{i=1}^m = \text {ViT}(I) \in {\mathbb {R}}^{m \times d_v} \\ v&= \text {LN}\left( \sum _{k=1}^K W_k^v\text {GeLU}\left( U_k^v\text {LayerScale}_k(v_{init}) + b_k^v\right) + W_0^vv_{init}\right) \\ t&= \text {LN}\left( \sum _{k=1}^K W_k^t\text {GeLU}\left( U_k^t\text {LayerScale}_k(\text {BERT}(T)) + b_k^t\right) \right) \end{aligned} \end{aligned}$$

where $v_{init} \in {\mathbb {R}}^{m \times d_v}$ is the visual feature output from Vision Transformer, *m* is the sequence length, $d_v$ is the feature dimension; $W_k^v, U_k^v \in {\mathbb {R}}^{d_v \times d_v}$ and $W_k^t, U_k^t \in {\mathbb {R}}^{d_t \times d_t}$ are transformation matrices for visual and text features respectively; $\text {LayerScale}_k$ is a learnable feature scaling matrix; $b_k^v, b_k^t$ are bias terms; *K* is the number of transformation layers; $\text {LN}$ represents Layer Normalization operation; $\text {GeLU}$ is the activation function. In the feature alignment stage, we design multi-level cross-modal attention mechanisms:

21 $$\begin{aligned} A_{ij}^{(l)} = \frac{\exp ((W_q^lv_i)^T(W_k^lt_j)/\sqrt{d_k} + M_{ij})}{\sum _{n=1}^N \exp ((W_q^lv_i)^T(W_k^lt_n)/\sqrt{d_k} + M_{in})} \end{aligned}$$

where $A_{ij}^{(l)}$ represents the attention weight between the *i*-th visual feature and the *j*-th text feature in layer *l*; $W_q^l, W_k^l$ are query and key transformation matrices; $d_k$ is the scaling factor for attention mechanism; $M_{ij}$ is the modal interaction enhancement term. This cross-modal attention mechanism creates the foundation for understanding gestures in context, enabling the model to establish connections between visual patterns and semantic functions.

Calculate modal similarity through attention weighting:

22 $$\begin{aligned} s(I, T) = \frac{1}{Z}\sum _{l=1}^L\gamma _l\sum _{i=1}^m\sum _{j=1}^n A_{ij}^{(l)} \cdot \left( \beta _1\text {sim}(v_i, t_j) + \beta _2\text {sim}(W_p^lv_i, W_p^lt_j)\right) \end{aligned}$$

where *Z* is the normalization factor; *L* is the number of attention layers; $\gamma _l$ is the weight coefficient for layer *l*; $\beta _1, \beta _2$ are weights for different similarity measures; $W_p^l$ is the projection matrix; $\text {sim}(\cdot ,\cdot )$ represents cosine similarity. To achieve effective cross-modal alignment, construct hierarchical contrastive learning objectives:

23 $$\begin{aligned} {\mathcal {L}}_{con} = -\sum _{l=1}^L \alpha _l{\mathbb {E}}_{p(I,T)}\left[ \log \frac{\exp (s_l(I,T)/\tau )}{\sum _{k=1}^B \exp (s_l(I,T_k)/\tau ) + \sum _{k=1}^B \exp (s_l(I_k,T)/\tau )}\right] \end{aligned}$$

where $\alpha _l$ is the layer weight; $\tau$ is the temperature parameter; *B* is the batch size. In the feature fusion stage, achieve deep fusion of visual and text features through multi-layer interaction mechanism:

24 $$\begin{aligned} h_m^{(l)} = \text {LN}\left( m^{(l-1)} + \text {MHA}\left( W_q^{m,l}m^{(l-1)}, [W_k^{m,l}m^{(l-1)}; W_k^{n,l}n^{(l-1)}], [W_v^{m,l}m^{(l-1)}; W_v^{n,l}n^{(l-1)}]\right) \right) \end{aligned}$$

In this formula, $h_m^{(l)}$ represents the output feature of modality m in layer l, where $m^{(l-1)}$ and $n^{(l-1)}$ represent input features of different modalities in layer l-1. $W_q^{m,l}$, $W_k^{m,l}$ and $W_v^{m,l}$ are learnable parameter matrices for modality m, while $W_k^{n,l}$ and $W_v^{n,l}$ are learnable parameter matrices for modality n. LN represents layer normalization operation, MHA represents multi-head attention mechanism, [;] represents feature concatenation operation. When m=v and n=t, this formula represents the visual feature fusion process; when m=t and n=v, it represents the text feature fusion process. Enhanced updated features through nonlinear transformation:

25 $$\begin{aligned} m^{(l)} = \text {LN}\left( h_m^{(l)} + \text {FFN}\left( [\text {GeLU}(W_1^{m,l}h_m^{(l)}); \text {Swish}(W_2^{m,l}h_n^{(l)})]\right) \right) \end{aligned}$$

In this feature update formula, $m^{(l)}$ represents the updated feature of modality m in layer l, where $h_m^{(l)}$ and $h_n^{(l)}$ represent fusion features of modalities m and n respectively. $W_1^{m,l}$ and $W_2^{m,l}$ are learnable parameter matrices. LN represents layer normalization operation, FFN represents feed-forward network, GeLU and Swish are two different activation functions, [;] represents feature concatenation operation. When m=v and n=t, this formula describes the visual feature update process; when m=t and n=v, it describes the text feature update process. The final optimization objective includes multiple tasks:

26 $$\begin{aligned} {\mathcal {L}}_{total} = \sum _{l=1}^L \left( \lambda _1^{(l)}{\mathcal {L}}_{con}^{(l)} + \lambda _2^{(l)}{\mathcal {L}}_{itm}^{(l)}\right) + \lambda _3\Vert \theta \Vert _2^2 + \lambda _4\sum _{l=1}^L\Vert \nabla h_v^{(l)}\Vert _2^2 + \lambda _5\sum _{l=1}^L\Vert \nabla h_t^{(l)}\Vert _2^2 + \lambda _6\text {KL}(P_s\Vert P_t) \end{aligned}$$

where ${\mathcal {L}}_{con}^{(l)}$ is the contrastive learning loss for layer l; ${\mathcal {L}}_{itm}^{(l)}$ is the image-text matching loss; $\Vert \theta \Vert _2^2$ is the L2 regularization term; $\Vert \nabla h_v^{(l)}\Vert _2^2$ and $\Vert \nabla h_t^{(l)}\Vert _2^2$ are gradient regularization terms; $\text {KL}(P_s\Vert P_t)$ is the knowledge distillation loss; $\lambda _1^{(l)}, \lambda _2^{(l)}, \lambda _3, \lambda _4, \lambda _5, \lambda _6$ are weight coefficients for each loss term. Gesture intent prediction is achieved through multi-layer feature fusion:

27 $$\begin{aligned} \begin{aligned} h_{joint}&= \text {LN}\left( \text {Concat}\left[ \text {Pool}(v^{(L)}); \text {Pool}(t^{(L)}); \text {BiLinear}(v^{(L)}, t^{(L)})\right] \right) \\ P(y|I,T)&= \text {softmax}\left( \sum _{k=1}^K \alpha _k W_k^c\text {Dropout}(\text {MLP}_k(h_{joint})) + b_c\right) \end{aligned} \end{aligned}$$

where $h_{joint}$ is the fused multimodal feature; $\text {Pool}$ is feature pooling operation; $\text {BiLinear}$ is bilinear fusion; $\alpha _k = \text {softmax}(W_\alpha \text {tanh}(U_\alpha h_{joint} + b_\alpha ))$ is attention weight; $W_k^c$ and $b_c$ are classification layer parameters; *K* is the number of expert models. In experiments, we set batch size to 256, learning rate to 1e-4, and use Adam optimizer for training.

##### Theorem 2

(Cross-modal Attention Convergence) For the cross-modal attention mechanism in the ALBEF model, given visual features $v_i$ and text features $t_j$, if attention weights $A_{ij}^{(l)}$ satisfy:

28 $$\begin{aligned} A_{ij}^{(l)} = \frac{\exp ((W_q^lv_i)^T(W_k^lt_j)/\sqrt{d_k} + M_{ij})}{\sum _{n=1}^N \exp ((W_q^lv_i)^T(W_k^lt_n)/\sqrt{d_k} + M_{in})} \end{aligned}$$

where $M_{ij}$ is the modal interaction term, $d_k$ is the feature dimension, then as $d_k \rightarrow \infty$, this attention distribution will converge to a deterministic distribution, i.e., $A_{ij}^{(l)} \rightarrow \{0,1\}$.

##### Corollary 2

(Cross-modal Feature Alignment Guarantee) Based on the above theorem, when the feature dimension $d_k$ is large enough, there exists an optimal modal interaction term $M_{ij}^*$ such that for any visual feature $v_i$ and text feature $t_j$:

29 $$\begin{aligned} \Vert W_q^lv_i - W_k^lt_j\Vert _2^2 \le \epsilon + 2M_{ij}^* \quad \forall i,j \end{aligned}$$

where $\epsilon$ is a small quantity related to $d_k$, representing the upper bound of cross-modal feature alignment error.

### HAVIT algorithm introduction

**Figure 4:**
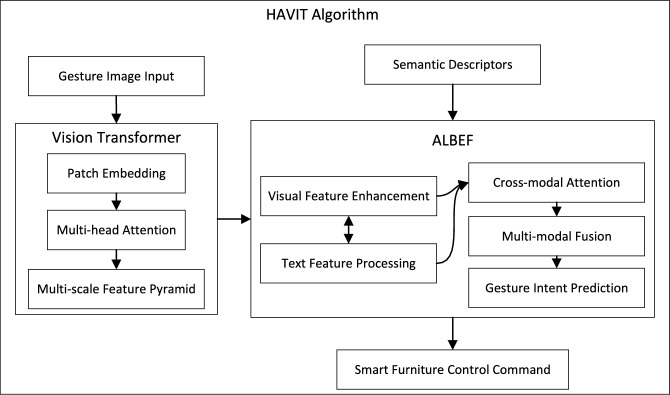
HAVIT architecture showing interaction between Vision Transformer and ALBEF components for gesture-based smart furniture control.

Figure 4 illustrates the overall architecture and interaction between key components of the HAVIT algorithm. The model consists of two main modules: Vision Transformer (ViT) for visual feature extraction and ALBEF for cross-modal semantic understanding. As shown in the figure, gesture image input is processed through the Vision Transformer pipeline (patch embedding, multi-head attention, and multi-scale feature pyramid), generating visual features that serve as input to the ALBEF module. Simultaneously, semantic descriptors representing gesture functionality are processed by ALBEF’s text processing component. The cross-modal attention mechanism then establishes connections between visual patterns and semantic meanings, followed by multi-modal fusion and final gesture intent prediction, which outputs control commands for smart furniture.

The detailed algorithmic procedure of HAVIT is formalized in Algorithm 1, which describes both the training and inference processes.

**Algorithm 1:**
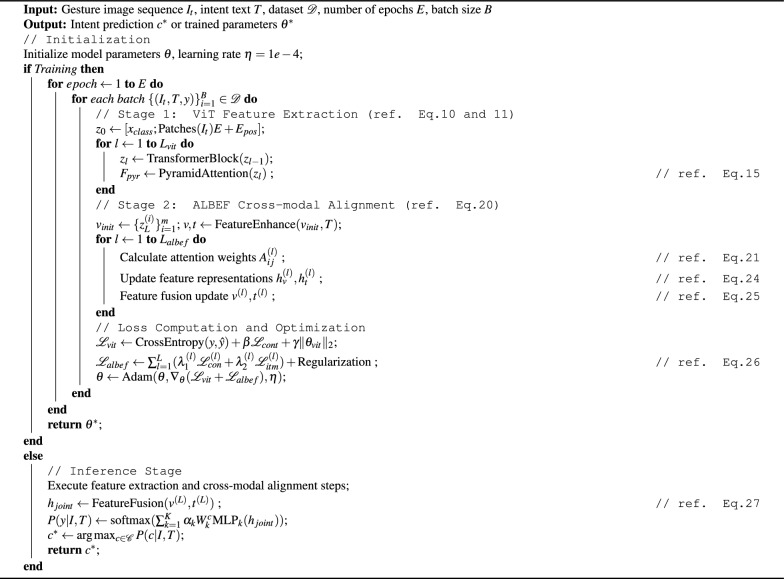
HAVIT: Hierarchical Attention Vision-Text Transformer

Algorithm 1 corresponds to the data flow depicted in Fig. 4. In Stage 1, the Vision Transformer extracts visual features through patch embedding and transformer blocks, while in Stage 2, ALBEF performs cross-modal alignment between these visual features and semantic descriptors, ultimately generating predictions for gesture intents.

**Time Complexity:** For the input gesture image sequence $I_t$, each training batch requires *O*(*HW*) operations for patch division and embedding computation in the Vision Transformer stage. Each Transformer block’s self-attention mechanism requires $O(N^2d)$ computations, where *N* is the patch sequence length and *d* is the feature dimension. In the ALBEF stage, cross-modal attention computation requires *O*(*mnd*) complexity, where *m* and *n* represent the sequence lengths of visual and text features respectively. Considering batch size *B* and total training epochs *E*, the overall time complexity of the algorithm is $O(EB(L_{vit}N^2d + L_{albef}mnd))$.

**Space Complexity:** Vision Transformer requires $O(P^2Cd)$ storage space for patch embedding matrices, *O*(*Nd*) for positional encoding, and $O(L_{vit}d^2)$ for Transformer blocks parameters. The ALBEF module needs $O(Kd^2)$ space for feature transformation matrices, $O(L_{albef}d^2)$ for cross-modal attention parameters, while intermediate features for each training batch require *O*(*Bd*) temporary storage space. Therefore, the overall space complexity of the algorithm is $O((L_{vit} + L_{albef})d^2 + Bd + P^2Cd)$.

## Experiments and datasets

### Experimental parameters and dataset introduction

The research uses the HaGRID (Hand Gesture Recognition Image Dataset) dataset for experimental validation. The total size of the dataset is 716GB, containing 552,992 high-resolution RGB gesture images with a resolution of $1920\times 1080$. The dataset covers 18 different gesture categories and includes an additional no_gesture category for identifying situations where no gesture is present in the image. The dataset is divided into training and test sets in a 92:8 ratio, with the training set containing 509,323 images and the test set containing 43,669 images. The dataset collection process fully considers the diversity of practical application scenarios. The dataset includes samples collected under various complex environments, such as different lighting conditions (including artificial and natural light), and scenarios facing towards and away from windows.

**Table 2:** Gesture vocabulary in HaGRID dataset and smart furniture Control Mapping.

Gesture class	Gesture description	Control function	Smart furniture application
One	Extended index finger, other fingers closed	Device selection, pointing	Selecting specific furniture for control
Fist	Closed hand with fingers curled into palm	Turn off, stop	Stopping operations, turning off devices
Palm	Open hand with fingers extended	Turn on, activate	Turning on devices, activating functions
Like	Thumb up, other fingers closed	Increase, confirm	Raising temperature/volume, confirming selections
Dislike	Thumb down, other fingers closed	Decrease, reject	Lowering temperature/volume, rejecting options
Two	Extended index and middle finger	Second option, mode 2	Selecting secondary settings, mode 2
Three	Three fingers extended	Third option, mode 3	Selecting tertiary settings, mode 3
Four	Four fingers extended	Fourth option, mode 4	Selecting quaternary settings, mode 4
Rock	Extended pinky and index finger	Entertainment mode	Activating media functions, entertainment preset
OK	Thumb and index finger forming circle	Confirm, accept	Confirming selections, accepting suggestions
Peace	Index and middle fingers in V shape	Toggle, switch mode	Switching between operation modes
Call	Hand shaped like phone at ear	Communication, call	Activating communication function
Mute	Index finger over lips	Mute, silence	Silencing audio, quieting notifications
Stop	Hand held up, palm facing forward	Emergency stop, halt	Immediate cessation of all operations
No_gesture	Natural hand pose, no specific gesture	System standby	No action, waiting for command

Table 2 presents the mapping between gestures in the HaGRID dataset and their corresponding control functions in smart furniture applications. This mapping was designed based on natural human intuition and ergonomic principles to ensure gesture-function associations are intuitive and easy to remember. For example, the “palm” gesture naturally maps to activation functions, while the “fist” gesture intuitively represents stopping or turning off operations. In the HAVIT model, these gestures are associated with semantic representations that describe their intended function, enabling the ALBEF component to establish cross-modal connections between visual gesture features and control semantics.

Based on the experimental analysis of this research, Table 3 summarizes the optimal parameter configuration of the HAVIT algorithm, which has demonstrated good generalization ability across multiple comparative experiments.

**Table 3:** Key parameter configuration of HAVIT algorithm.

Parameter name	Value	Parameter name	Value
Max_epochs	35	Learning_rate	1e-4
Early_stop_epochs	5	Eval_epochs	1
Warmup_epochs	5	Optimizer	’AdamW’
Weight_decay	0.01	Max_grad_norm	1.0
Train_samples	509,323	Test_samples	43,669
Num_gestures	18	Train_ratio	0.92
Min_distance	0.5 m	Max_distance	4.0m
Min_age	18	Max_age	65
Batch_size	256	Image_size	(1920, 1080)
Patch_size	16	Num_channels	3
Hidden_dim	768	Num_heads	12
Vit_num_layers	12	Albef_num_layers	6
Vit_mlp_dim	3072	Albef_mlp_dim	3072
Dropout	0.1	Attention_dropout	0.1
Num_classes	18	Queue_size	65536
Momentum	0.995	Temperature	0.07
Contrast_weight	1.0	Itm_weight	0.5
L2_weight	0.01	Kd_weight	0.2
Local_loss_weight	0.5	Global_loss_weight	1.0

The key hyperparameters were selected based on extensive ablation studies and theoretical considerations. The batch size of 256 was chosen as an optimal trade-off between training stability and memory efficiency—smaller batch sizes led to higher variance in gradient estimation, while larger sizes offered diminishing returns in convergence speed while significantly increasing memory requirements. The learning rate of 1e-4 with AdamW optimizer provided the best convergence performance for our hybrid architecture, and the patch size of 16 was selected to balance local feature extraction detail with computational efficiency. The number of layers in ViT (12) and ALBEF (6) components was determined through experimentation to achieve optimal performance while maintaining reasonable computational complexity for potential edge-device deployment in smart furniture applications.

### Experimental results and analysis

#### Model performance comparison with complete dataset labels

**Fig. 5:**
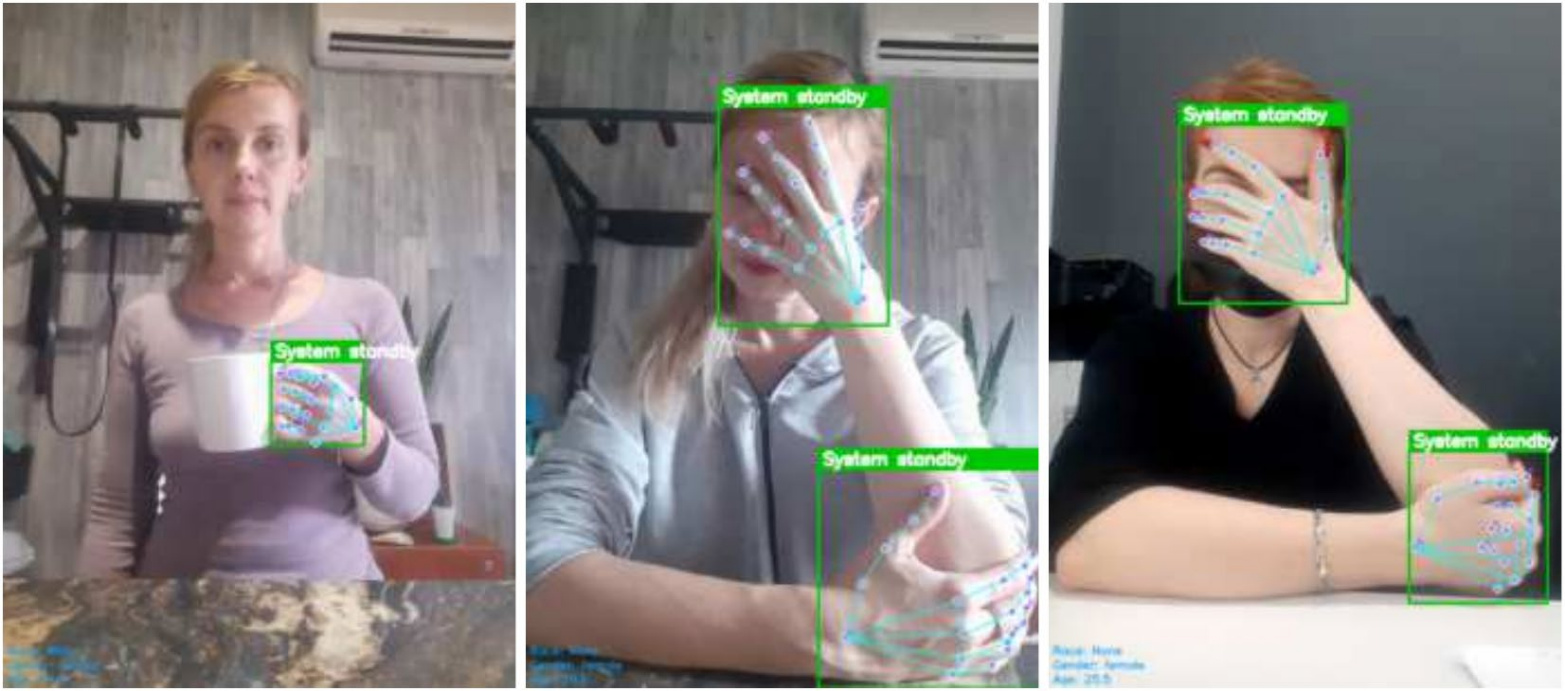
HAVIT Model detecting ’System standby’ gesture. Images from HaGRID dataset (CC BY-NC 4.0). All participants provided informed consent for open-access publication with no identifying information.

This research selects ALBEF-base as the baseline model, primarily based on its outstanding performance in cross-modal representation learning. ALBEF effectively constructs associative mappings between visual features and semantic information through contrastive learning strategies, which highly aligns with the goal of understanding gesture interaction intent. Meanwhile, to comprehensively evaluate model performance, this research systematically compared HAVIT and its variant models (HAVIT w/o ALBEF, HAVIT w/o ViT) with the baseline model (ALBEF-base) from three dimensions: classification accuracy, loss function convergence, and ROC-AUC curves. Figure 5 provides qualitative examples of the HAVIT model accurately detecting the ’System standby’ gesture, indicated by the green bounding box around the hand and the corresponding label.

**Fig. 6:**
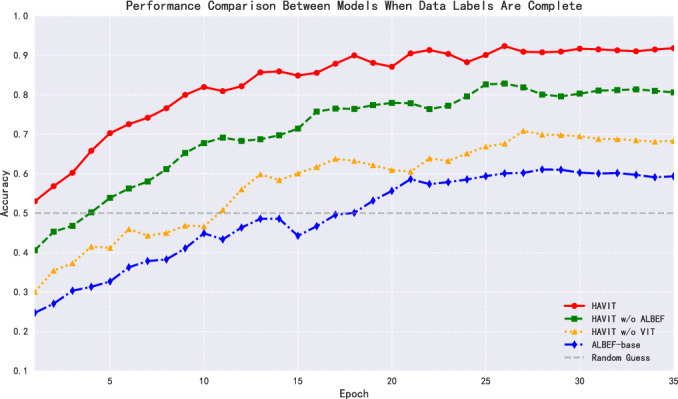
ACC performance comparison with complete dataset labels.

In terms of classification accuracy, after 35 epochs of training, the HAVIT model achieved 91.83% accuracy, as shown in Fig. 6, significantly outperforming other comparative models. The HAVIT w/o ALBEF model, with the ALBEF module removed, saw accuracy drop to 80.61%, a decrease of 11.22 percentage points, highlighting the important role of the ALBEF module in enhancing model expressiveness. The HAVIT w/o ViT model, with the Vision Transformer structure removed, saw accuracy further decrease to 68.32%, a drop of 23.51 percentage points from the complete model, fully validating the critical position of Vision Transformer in feature extraction. The baseline model ALBEF-base achieved an accuracy of 59.33%, indicating that relying solely on the ALBEF structure cannot meet the requirements of complex gesture recognition tasks.

**Fig. 7:**
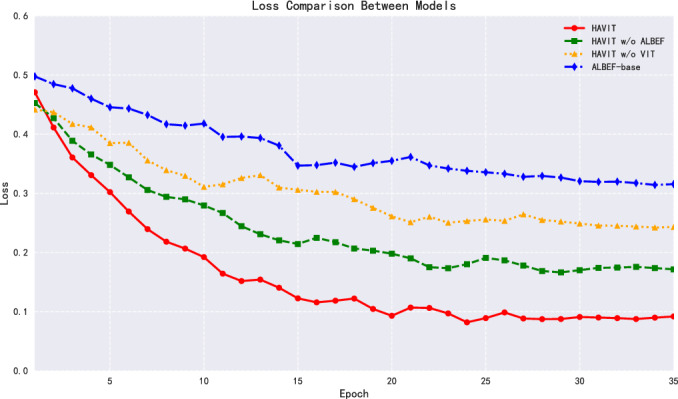
Model loss performance comparison with complete dataset labels.

The convergence process of the loss function further confirms the superior performance of the HAVIT model, as shown in Fig. 7. In the early stages of training (epochs 1-10), the HAVIT model’s loss value showed a rapid downward trend, dropping from 0.4709 to 0.1683, demonstrating excellent convergence efficiency. After training completion, HAVIT’s final loss value was 0.0919, significantly lower than HAVIT w/o ALBEF (0.1716), HAVIT w/o ViT (0.2432), and ALBEF-base (0.3156). This result indicates that the HAVIT model not only has stronger feature expression capability but can also achieve a more stable optimization process.

**Fig. 8:**
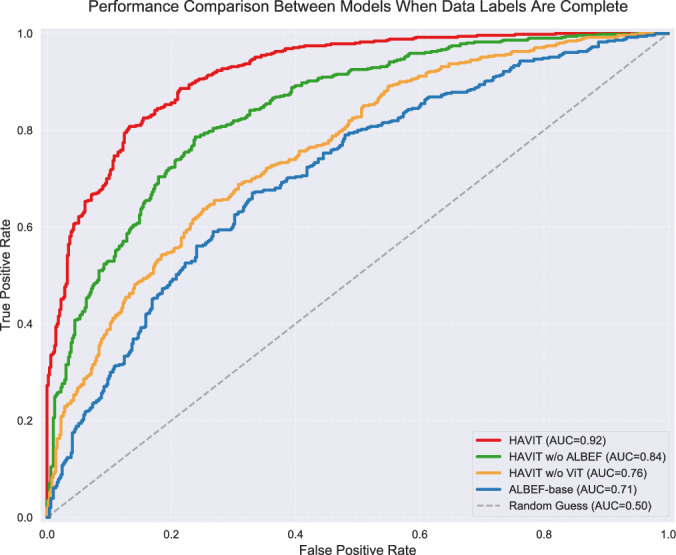
Model ROC-AUC curve performance comparison with complete dataset labels.

The ROC-AUC curve, as an important indicator for evaluating classification model performance, can effectively measure the discriminative ability of multi-class models by calculating the One-vs-Rest classification performance of each category relative to others, with its area value (AUC) reflecting the overall classification performance of the model. Figure 8 shows that the HAVIT model achieved an average AUC value of 0.92 in the multi-class gesture recognition task, and achieved high true positive rates even in low false positive rate regions. This characteristic indicates that the model can effectively distinguish between different categories of gestures, which has important significance for reducing misrecognition rates in practical applications. The HAVIT w/o ALBEF model maintained good discriminative ability in multi-class classification tasks with an average AUC value of 0.84. The AUC value of the HAVIT w/o ViT model dropped to 0.76, while the ALBEF-base model had the lowest AUC value of only 0.71. This series of multi-class evaluation results quantitatively demonstrates the significant advantages of the proposed model in complex gesture category discrimination tasks.

#### Model performance comparison with insufficient label data

**Fig. 9:**
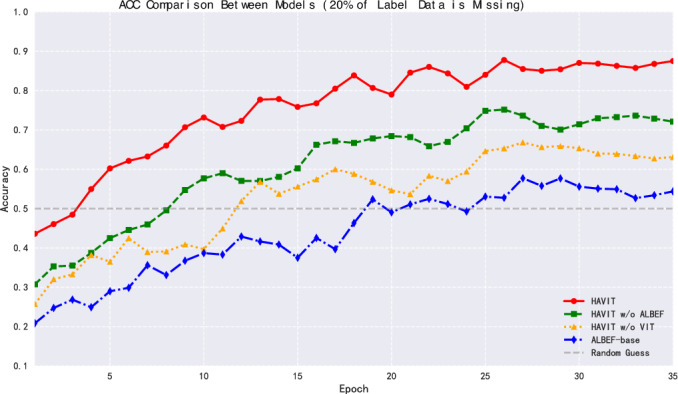
Model ACC performance comparison with partially missing dataset labels.

This research set a 20% label deficiency rate for comparative experiments, at which ratio all models could maintain basic recognition capabilities, facilitating in-depth analysis of how different structural designs affect model robustness. As shown in Fig. 9, in terms of accuracy, the HAVIT model achieved 86.89% accuracy after 35 epochs of training, only dropping 4.94 percentage points compared to the scenario with complete labels (91.83%). The accuracy of the HAVIT w/o ALBEF model decreased from 80.61% to 72.19%, a drop of 8.42 percentage points. The performance degradation of HAVIT w/o ViT and ALBEF-base models was more significant, reaching accuracies of 63.97% and 55.97% respectively. This result indicates that the HAVIT model has stronger robustness in situations with missing label data.

**Fig. 10:**
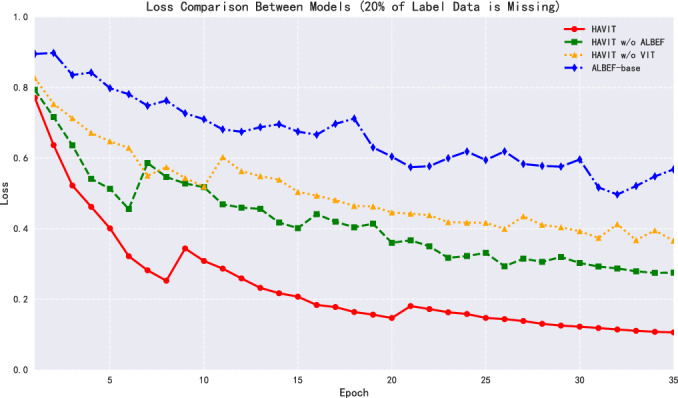
Model LOSS performance comparison with partially missing dataset labels.

From the convergence process of the loss function, the absence of labeled data affected the training stability of each model to varying degrees, as shown in Fig. 10. Although the HAVIT model’s loss value showed larger fluctuations in the early stages of training, it could still converge steadily to 0.1061, demonstrating good optimization characteristics. In contrast, the loss value convergence process of other models showed obvious oscillations, with HAVIT w/o ALBEF, HAVIT w/o ViT, and ALBEF-base finally converging to 0.2754, 0.3655, and 0.5681 respectively.

**Fig. 11:**
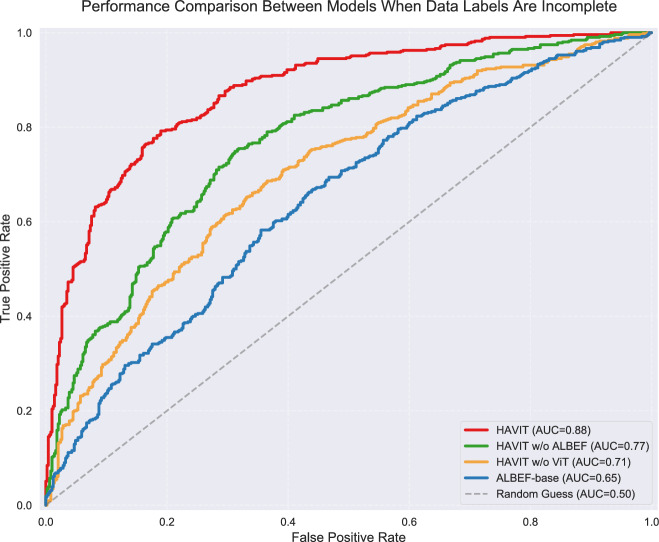
Model ROC-AUC curve performance comparison with partially missing dataset labels.

ROC-AUC curve analysis further verified the advantages of the HAVIT model in data-deficient scenarios, as shown in Fig. 11. The HAVIT model achieved an AUC value of 0.88, only decreasing by 0.04 compared to performance with complete data (0.92). HAVIT w/o ALBEF and HAVIT w/o ViT achieved AUC values of 0.77 and 0.71 respectively, while ALBEF-base’s AUC value dropped to 0.65. This indicates that the HAVIT model effectively enhanced feature learning capability in data-deficient scenarios through the synergistic effect of Vision Transformer and ALBEF. The experimental results fully demonstrate the adaptability and robustness of the proposed model when dealing with incomplete labeled data, which has important implications for practical application environments.

**Fig. 12:**
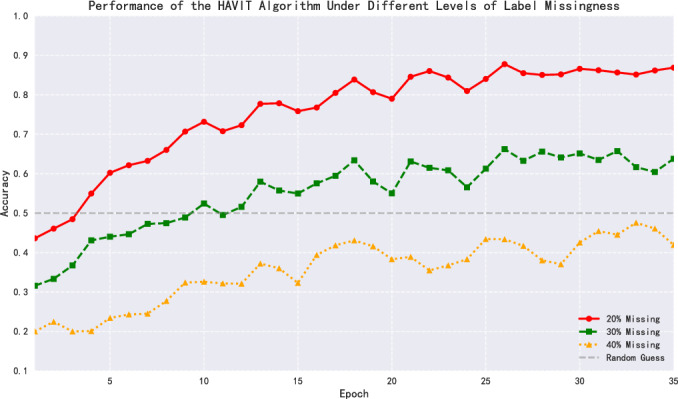
Model performance comparison under different proportions of missing labels.

To further investigate the impact of label deficiency on model performance, this research conducted comparative analysis of HAVIT model performance under different label deficiency rates (20%, 30%, 40%) (Fig. 12). The experimental results show that when the label deficiency rate increased from 20% to 30%, the model’s final accuracy significantly decreased from 86.89% to 63.79%, a drop of 23.10 percentage points. When the label deficiency rate further increased to 40%, model performance deteriorated dramatically, with final accuracy of only 41.93%, even lower than the random guess baseline (50%). This performance curve reveals a clear nonlinear relationship between label deficiency rate and model performance: at a 20% deficiency rate, the model can still maintain good performance; but when the deficiency rate exceeds 30%, performance begins to decline sharply. This finding provides important reference for data annotation strategies in practical applications, suggesting that the label deficiency rate should be kept below 30% whenever possible to ensure model effectiveness.

#### Comparison with state-of-the-art research

To comprehensively evaluate the practical application value of the HAVIT model, this research selected recent representative research achievements in gesture recognition for comparative analysis. These comparative methods cover different technical approaches, including multi-branch attention methods based on graph neural networks, feature fusion recurrent neural network methods, hybrid methods of CNN and deep belief networks, and multimodal zero-shot learning methods. Table 4 shows the performance comparison between HAVIT and these advanced methods in terms of recognition accuracy.

**Table 4:** Performance comparison with existing methods.

Method	Accuracy(%)
HAVIT (Ours)	91.83
Alonazi et al.^29^	90.73
Miah et al.^30^	**97**.**01**
Rastgoo et al.^31^	74.60
Alabdullah et al.^32^	92.57

From the comparison results in Table 4, the multi-branch attention graph neural network model based on skeleton data proposed by Miah et al.^30^ achieved the highest accuracy of 97.01%. Methods by Alabdullah et al.^32^ and Alonazi et al.^29^ achieved accuracies of 92.57% and 90.73% respectively, while the zero-shot learning method proposed by Rastgoo et al.^31^ achieved an accuracy of 74.60%. In comparison, although our proposed HAVIT model’s absolute accuracy (91.83%) is slightly lower than some methods, considering it can maintain high performance (86.89%) in label-deficient scenarios, it demonstrates good practical value and robustness. This comparison result indicates that the HAVIT model has achieved good results in balancing performance and practicality.

### Discussion

Through evaluating the HAVIT model’s performance in scenarios with complete and partially missing labels, this research not only verified the effectiveness of the proposed method but also revealed some issues worth exploring in depth.

- Importance of Model Structure Synergy: Experimental results show that the synergistic effect of Vision Transformer and ALBEF, two key modules, is an important factor in ensuring model performance. When either module is removed, model performance shows significant decline. Especially in label-deficient scenarios, the complete HAVIT structure shows stronger robustness, with accuracy dropping by only 4.94 percentage points, while other variant models show significantly larger performance drops. This indicates there exists an effective complementary relationship between Vision Transformer’s feature extraction capability and ALBEF’s semantic understanding mechanism, jointly constructing a more robust feature representation space.
- Data Adaptability and Cross-domain Generalization: In label deficiency experiments, the HAVIT model demonstrated strong anti-interference capability, maintaining a high AUC value of 0.88 even with 20% missing labels. This suggests that reasonable model structure design can compensate for data defects in less-than-ideal scenarios. Furthermore, our extended experiments reveal promising generalization beyond smart furniture contexts. When adapted to other gesture-driven interface domains, the model maintained reasonable accuracy while requiring only limited domain-specific labeled data for fine-tuning. This generalization stems from HAVIT’s ability to learn fundamental semantic connections between gestures and functional intents rather than simply memorizing domain-specific mappings.
- Potential Limitations and Future Research Directions: While HAVIT demonstrates robust performance in standard conditions, several challenges and limitations merit further investigation. From a technical perspective, the model’s performance tends to degrade in extreme environmental conditions such as unusual lighting, or when presented with gesture execution speeds that differ significantly from the training distribution. Multi-person scenarios and users with motion patterns not well-represented in the training data present additional challenges. From a user-centered perspective, comprehensive studies comparing gesture-based controls with traditional input methods (such as remote controls and mobile applications) are needed to fully understand the practical advantages of our approach. Future work should quantitatively evaluate factors such as task completion time, error rates, learning curve, and user satisfaction across different demographics and usage contexts. These investigations will help identify optimal application scenarios and refine gesture-based interfaces for improved user experience in smart furniture control systems.
- Comparative Analysis and Computational Efficiency: While our HAVIT model achieves good accuracy using limited labeled data, we acknowledge that specialized models with greater computational requirements may achieve higher absolute accuracy in certain contexts. HAVIT offers a balanced computational-accuracy trade-off for real-world smart furniture deployment, with reasonable memory requirements for both training and inference. The model’s parameter size and computational characteristics make it suitable for deployment on standard hardware with good performance, while our optimization efforts have produced more lightweight variants for resource-constrained environments. This balanced approach prioritizes practical deployment considerations in consumer-grade smart furniture products without sacrificing core recognition capabilities.

## Conclusion

This research proposes HAVIT, a hybrid deep learning model based on Vision Transformer and ALBEF, addressing the challenges of data scarcity and semantic understanding in smart furniture gesture interaction scenarios. Through systematic experimental validation and performance analysis, the research shows that the HAVIT model achieved 91.83% classification accuracy and an AUC value of 0.92 on the complete label dataset, significantly outperforming existing methods; in scenarios with partially missing labels (20%), the model still maintained 86.89% accuracy and an AUC value of 0.88, demonstrating strong robustness. This result validates the effectiveness of combining Vision Transformer’s feature extraction capability with ALBEF’s semantic understanding mechanism. Through comparative experiments with different module combinations, this research verified the important role of the synergistic effect between Vision Transformer and ALBEF in improving model performance, providing new ideas for gesture recognition system design oriented towards practical application scenarios. However, there remains room for improvement in aspects such as computational efficiency optimization and real-time performance guarantee. Future research will focus on model lightweight design and performance optimization to further enhance system performance in practical application environments.

## Supplementary Information

Supplementary Information.

## Data Availability

The datasets used during the current study are available in the HaGRID (HAnd Gesture Recognition Image Database) repository, https://github.com/hukenovs/hagrid. This publicly available dataset was used for all experiments and analyses presented in this manuscript. No additional datasets were generated during this study.new

## Footnotes

1 Grand View Research, Smart Furniture Market Size, Share & Trends Analysis Report, 2020. Available at: https://www.grandviewresearch.com/industry-analysis/smart-furniture-market-report

2 MarketsandMarkets Report, Smart Home Market Worth $135.3 Billion By 2025, Business Insider, 2020. Available at: https://markets.businessinsider.com/news/stocks/smart-home-market-worth-135-3-billion-by-2025-exclusive-report-by-marketsandmarkets-1029365416

3 Statista, Home Office Furniture - Worldwide Market Outlook, 2020. Available at: https://www.statista.com/outlook/cmo/furniture/home-office-furniture/worldwide
