# Supplementary material for: HAVIT: research on vision-language gesture interaction mechanism for smart furniture
Source: Sci Rep. 2025 Jul 28;15:27423. doi: 10.1038/s41598-025-10758-9 (PMC12304170; doi:10.1038/s41598-025-10758-9)
Supplement: Supplementary file 1 — Supplementary Information. [file 41598_2025_10758_MOESM1_ESM.pdf]

5 Appendix: Theorems, Corollaries, and Proofs

Theorem 1 (Multi-scale Feature Representation Theorem) *For any gesture image sequence I_t and its feature representation z_l in Vision Transformer, there exists an optimal set of scale weights $\{w_s^*\}_{s=1}^S$, such that the multi-scale feature pyramid representation satisfies:*

$$\|\mathscr{F}(I_t) - \sum_{s=1}^{S} w_s^* \cdot Attention(Q_s, K_s, V_s)\|_2 \leq \varepsilon$$

where $\mathscr{F}(I_t)$ is the ideal feature representation, $\varepsilon > 0$ is any small positive number, and w_s^* is determined by the statistical characteristics of the feature distribution.

Proof 1 *Consider feature representation $\mathscr{F}(I_t)$ in the complete separable metric space $\mathscr{H}$ with inner product $\langle \cdot, \cdot \rangle$. Given the completeness and separability properties of $\mathscr{H}$, we can construct an orthonormal basis $\{\phi_s\}_{s=1}^{\infty}$ through Gram-Schmidt orthogonalization process such that:*

$$\mathscr{F}(I_t) = \sum_{s=1}^{\infty} \alpha_s \phi_s, \quad \text{where } \alpha_s = \langle \mathscr{F}(I_t), \phi_s \rangle$$

For the attention mechanism in Vision Transformer, at each scale s, we define:

$$A_s = Attention(Q_s, K_s, V_s) = softmax\left(\frac{Q_s K_s^T}{\sqrt{d_k}}\right) V_s$$

The attention output A_s can be decomposed into basis functions:

$$A_s = \sum_{i=1}^{N_s} \beta_{s,i} \psi_{s,i}, \quad \text{where } \beta_{s,i} = \langle A_s, \psi_{s,i} \rangle$$

By the universal approximation property of attention mechanisms, there exists a linear transformation T_s such that:

$$\|T_s(\psi_{s,i}) - \phi_i\|_2 \leq \frac{\varepsilon}{2S\|\beta_{s,i}\|_2}, \quad \forall i \leq N_s$$

For the multi-head attention structure with H heads:

$$MultiHead(Q, K, V) = Concat(head_1, ..., head_H) W^O$$

where each head is computed as:

$$head_h = Attention(QW_Q^h, KW_K^h, VW_V^h)$$

The optimal weights w_s^* are obtained by minimizing:

$$w_s^* = \arg\min_{w_s} \|\mathscr{F}(I_t) - \sum_{s=1}^{S} w_s A_s\|_2$$

By the triangle inequality:

$$\|\mathscr{F}(I_t) - \sum_{s=1}^{S} w_s^* A_s\|_2 \leq \|\sum_{s=S+1}^{\infty} \alpha_s \phi_s\|_2 + \|\sum_{s=1}^{S} (\alpha_s \phi_s - w_s^* A_s)\|_2$$

Using the Cauchy-Schwarz inequality:

$$\|\sum_{s=S+1}^{\infty}\alpha_s\phi_s\|_2 \le \left(\sum_{s=S+1}^{\infty}|\alpha_s|^2\right)^{1/2} \le \frac{\varepsilon}{2}$$

For the second term:

$$\|\sum_{s=1}^{S}(\alpha_s\phi_s - w_s^* A_s)\|_2 \le \sum_{s=1}^{S}\|\alpha_s\phi_s - w_s^* A_s\|_2 \le \frac{\varepsilon}{2}$$

Therefore:

$$\|\mathscr{F}(I_t) - \sum_{s=1}^{S} w_s^* A_s\|_2 \le \varepsilon$$

The error bound is achieved through the optimal selection of scale weights and the universal approximation property of the attention mechanism.

Corollary 1 (Uncertainty Bound of Feature Representation) *Based on the Multi-scale Feature Representation Theorem, for any two different gesture samples I_t^1 and I_t^2, the difference in their feature representations satisfies:*

$$\|f(I_t^1) - f(I_t^2)\|_2 \ge \delta\|I_t^1 - I_t^2\|_2 - 2\varepsilon$$

where $f(\cdot)$ represents features extracted through multi-scale Vision Transformer, and $\delta > 0$ is a constant related to the model architecture.

Proof 2 *First, let's express the feature representation $f(I_t)$ in terms of the multi-scale attention mechanism:*

$$f(I_t) = \sum_{s=1}^{S} w_s^* \cdot Attention(Q_s, K_s, V_s)$$

For any input I_t^k ($k = 1, 2$), the feature representation error is bounded by:

$$\|f(I_t^k) - \mathscr{F}(I_t^k)\|_2 \le \varepsilon$$

The attention mechanism at each scale can be decomposed as:

$$Attention(Q_s, K_s, V_s) = softmax\left(\frac{Q_s K_s^T}{\sqrt{d_k}}\right)V_s$$

Let's define the attention difference for two inputs:

$$\Delta A_s = Attention(Q_s^1, K_s^1, V_s^1) - Attention(Q_s^2, K_s^2, V_s^2)$$

By the Lipschitz property of softmax:

$$\|\Delta A_s\|_2 \le L_s\|I_t^1 - I_t^2\|_2$$

where L_s is the Lipschitz constant for scale s. Therefore:

$$\|f(I_t^1) - f(I_t^2)\|_2 = \left\|\sum_{s=1}^{S} w_s^* \Delta A_s\right\|_2$$

Using the triangle inequality:

$$
\begin{aligned}
\|f(I_t^1) - f(I_t^2)\|_2 &\geq \|\mathscr{F}(I_t^1) - \mathscr{F}(I_t^2)\|_2 - \|f(I_t^1) - \mathscr{F}(I_t^1)\|_2 - \|f(I_t^2) - \mathscr{F}(I_t^2)\|_2 \\
&\geq \|\mathscr{F}(I_t^1) - \mathscr{F}(I_t^2)\|_2 - 2\varepsilon
\end{aligned}
$$

By the mean value theorem and the properties of the Jacobian matrix:

$$
\|\mathscr{F}(I_t^1) - \mathscr{F}(I_t^2)\|_2 \geq \sigma_{\min}\left(\frac{\partial \mathscr{F}}{\partial I_t}\right) \|I_t^1 - I_t^2\|_2
$$

Define:

$$
\delta = \min_{I_t} \sigma_{\min}\left(\frac{\partial \mathscr{F}}{\partial I_t}\right)
$$

The Jacobian of the attention mechanism can be expressed as:

$$
\frac{\partial A_s}{\partial I_t} = \frac{\partial}{\partial I_t}\left[softmax\left(\frac{Q_s K_s^T}{\sqrt{d_k}}\right) V_s\right]
$$

The minimum singular value satisfies:

$$
\sigma_{\min}\left(\frac{\partial A_s}{\partial I_t}\right) \geq \frac{1}{\sqrt{d_k}} \min_{i,j} \left|\frac{\partial (Q_s K_s^T)_{ij}}{\partial I_t}\right|
$$

For the multi-head attention structure:

$$
\sigma_{\min}\left(\frac{\partial MultiHead}{\partial I_t}\right) \geq \min_h \sigma_{\min}\left(\frac{\partial head_h}{\partial I_t}\right)
$$

Combining these inequalities:

$$
\begin{aligned}
\|f(I_t^1) - f(I_t^2)\|_2 &\geq \delta \|I_t^1 - I_t^2\|_2 - 2\varepsilon \\
&= \min_{I_t} \sigma_{\min}\left(\frac{\partial \mathscr{F}}{\partial I_t}\right) \|I_t^1 - I_t^2\|_2 - 2\varepsilon
\end{aligned}
$$

The lower bound on the feature representation difference can be further refined using the properties of the attention layers:

$$
\delta \geq \min_{s,h} \left\{\frac{1}{\sqrt{d_k}} \sigma_{\min}(W_h^O) \min_{i,j} \left|\frac{\partial (Q_s K_s^T)_{ij}}{\partial I_t}\right|\right\}
$$

This completes the proof by establishing a precise relationship between input differences and feature representation differences, with explicit consideration of the attention mechanism's architecture and the approximation error ε.

Corollary 2 (Uncertainty Bound of Feature Representation) *Based on the Multi-scale Feature Representation Theorem, for any two different gesture samples I_t^1 and I_t^2, the difference in their feature representations satisfies:*

$$
\|f(I_t^1) - f(I_t^2)\|_2 \geq \delta \|I_t^1 - I_t^2\|_2 - 2\varepsilon
$$

where $f(\cdot)$ represents features extracted through multi-scale Vision Transformer, and $\delta > 0$ is a constant related to the model architecture.

Proof 3 *First, let's express the feature representation $f(I_t)$ in terms of the multi-scale attention mechanism:*

$$f(I_t) = \sum_{s=1}^{S} w_s^* \cdot Attention(Q_s, K_s, V_s)$$

For any input I_t^k ($k = 1,2$), the feature representation error is bounded by:

$$\|f(I_t^k) - \mathscr{F}(I_t^k)\|_2 \leq \varepsilon$$

The attention mechanism at each scale can be decomposed as:

$$Attention(Q_s, K_s, V_s) = softmax\left(\frac{Q_s K_s^T}{\sqrt{d_k}}\right) V_s$$

Let's define the attention difference for two inputs:

$$\Delta A_s = Attention(Q_s^1, K_s^1, V_s^1) - Attention(Q_s^2, K_s^2, V_s^2)$$

By the Lipschitz property of softmax:

$$\|\Delta A_s\|_2 \leq L_s \|I_t^1 - I_t^2\|_2$$

where L_s is the Lipschitz constant for scale s. Therefore:

$$\|f(I_t^1) - f(I_t^2)\|_2 = \left\| \sum_{s=1}^{S} w_s^* \Delta A_s \right\|_2$$

Using the triangle inequality:

$$\begin{aligned} \|f(I_t^1) - f(I_t^2)\|_2 &\geq \|\mathscr{F}(I_t^1) - \mathscr{F}(I_t^2)\|_2 - \|f(I_t^1) - \mathscr{F}(I_t^1)\|_2 - \|f(I_t^2) - \mathscr{F}(I_t^2)\|_2 \\ &\geq \|\mathscr{F}(I_t^1) - \mathscr{F}(I_t^2)\|_2 - 2\varepsilon \end{aligned}$$

By the mean value theorem and the properties of the Jacobian matrix:

$$\|\mathscr{F}(I_t^1) - \mathscr{F}(I_t^2)\|_2 \geq \sigma_{\min}\left(\frac{\partial \mathscr{F}}{\partial I_t}\right) \|I_t^1 - I_t^2\|_2$$

Define:

$$\delta = \min_{I_t} \sigma_{\min}\left(\frac{\partial \mathscr{F}}{\partial I_t}\right)$$

The Jacobian of the attention mechanism can be expressed as:

$$\frac{\partial A_s}{\partial I_t} = \frac{\partial}{\partial I_t}\left[softmax\left(\frac{Q_s K_s^T}{\sqrt{d_k}}\right) V_s\right]$$

The minimum singular value satisfies:

$$\sigma_{\min}\left(\frac{\partial A_s}{\partial I_t}\right) \geq \frac{1}{\sqrt{d_k}} \min_{i,j} \left| \frac{\partial (Q_s K_s^T)_{ij}}{\partial I_t} \right|$$

For the multi-head attention structure:

$$\sigma_{\min}\left(\frac{\partial MultiHead}{\partial I_t}\right) \geq \min_h \sigma_{\min}\left(\frac{\partial head_h}{\partial I_t}\right)$$

Combining these inequalities:

$$\begin{aligned}\|f(I_t^1) - f(I_t^2)\|_2 &\geq \delta \|I_t^1 - I_t^2\|_2 - 2\varepsilon \\ &= \min_{I_t} \sigma_{\min}\left(\frac{\partial \mathscr{F}}{\partial I_t}\right) \|I_t^1 - I_t^2\|_2 - 2\varepsilon\end{aligned}$$

The lower bound on the feature representation difference can be further refined using the properties of the attention layers:

$$\delta \geq \min_{s,h}\left\{\frac{1}{\sqrt{d_k}} \sigma_{\min}(W_h^O) \min_{i,j}\left|\frac{\partial (Q_s K_s^T)_{ij}}{\partial I_t}\right|\right\}$$

This completes the proof by establishing a precise relationship between input differences and feature representation differences, with explicit consideration of the attention mechanism's architecture and the approximation error ε.

Theorem 2 (Cross-modal Attention Convergence) *For the cross-modal attention mechanism in the ALBEF model, given visual features v_i and text features t_j, if attention weights $A_{ij}^{(l)}$ satisfy:*

$$A_{ij}^{(l)} = \frac{\exp((W_q^l v_i)^T (W_k^l t_j)/\sqrt{d_k} + M_{ij})}{\sum_{n=1}^N \exp((W_q^l v_i)^T (W_k^l t_n)/\sqrt{d_k} + M_{in})}$$

where M_{ij} is the modal interaction term, d_k is the feature dimension, then as $d_k \to \infty$, this attention distribution will converge to a deterministic distribution, i.e., $A_{ij}^{(l)} \to \{0,1\}$.

Proof 4 *To establish the convergence properties of the attention mechanism, we first decompose and analyze its constituent components. The attention mechanism can be formalized through the following mathematical constructions:Define the attention score before softmax:*

$$S_{ij} = \frac{(W_q^l v_i)^T (W_k^l t_j)}{\sqrt{d_k}} + M_{ij}$$

The query projection can be decomposed as:

$$W_q^l v_i = \sum_{p=1}^{d_k} w_{qp}^l v_{ip}$$

The key projection can be decomposed as:

$$W_k^l t_j = \sum_{p=1}^{d_k} w_{kp}^l t_{jp}$$

The dot product can be expressed as:

$$(W_q^l v_i)^T (W_k^l t_j) = \sum_{p=1}^{d_k} \sum_{q=1}^{d_k} w_{qp}^l w_{kq}^l v_{ip} t_{jp}$$

Define the normalized dot product:

$$Z_{ij} = \frac{(W_q^l v_i)^T (W_k^l t_j)}{\sqrt{d_k}}$$

By the Central Limit Theorem, as $d_k \to \infty$:

$$Z_{ij} \sim \mathcal{N}(\mu_{ij}, \sigma_{ij}^2/d_k)$$

The mean can be computed as:

$$\mu_{ij} = \mathbb{E}[Z_{ij}] = \frac{1}{\sqrt{d_k}} \sum_{p=1}^{d_k} \mathbb{E}[w_{qp}^l w_{kp}^l]\mathbb{E}[v_{ip} t_{jp}]$$

The variance can be expressed as:

$$\sigma_{ij}^2 = Var[Z_{ij}] = \frac{1}{d_k} \sum_{p=1}^{d_k} Var[w_{qp}^l w_{kp}^l v_{ip} t_{jp}]$$

For the modal interaction term, we can decompose it as:

$$M_{ij} = MLP\left(\left[\frac{v_i^T t_j}{\|v_i\|\|t_j\|}; ReLU(W_1^m[v_i; t_j] + b_1^m); cos(v_i, t_j)\right]\right)$$

The cosine similarity component:

$$cos(v_i, t_j) = \frac{v_i^T t_j}{\|v_i\|\|t_j\|} = \frac{\sum_{p=1}^{d_k} v_{ip} t_{jp}}{\sqrt{\sum_{p=1}^{d_k} v_{ip}^2}\sqrt{\sum_{p=1}^{d_k} t_{jp}^2}}$$

The attention weight distribution can be rewritten as:

$$A_{ij}^{(l)} = \frac{\exp(\mathcal{N}(\mu_{ij}, \sigma_{ij}^2/d_k) + M_{ij})}{\sum_{n=1}^{N} \exp(\mathcal{N}(\mu_{in}, \sigma_{in}^2/d_k) + M_{in})}$$

As $d_k \to \infty$, we have:

$$\lim_{d_k \to \infty} \frac{\sigma_{ij}^2}{d_k} = 0$$

Therefore:

$$\lim_{d_k \to \infty} A_{ij}^{(l)} = \frac{\exp(\mu_{ij} + M_{ij})}{\sum_{n=1}^{N} \exp(\mu_{in} + M_{in})}$$

Define the maximum attention score:

$$j^* = \arg\max_j (\mu_{ij} + M_{ij})$$

Then for the maximum attention weight:

$$\lim_{d_k \to \infty} A_{ij^*}^{(l)} = 1$$

For all other attention weights:

$$\lim_{d_k \to \infty} A_{ij}^{(l)} = 0, \quad \forall j \neq j^*$$

The convergence rate can be bounded by:

$$|A_{ij}^{(l)} - \mathbb{I}(j = j^*)| \leq C \exp(-d_k(\mu_{ij^*} + M_{ij^*} - \mu_{ij} - M_{ij})^2)$$

where C is a constant and $\mathbb{I}$ is the indicator function.

Corollary 3 (Cross-modal Feature Alignment Guarantee) *Based on the above theorem, when the feature dimension d_k is large enough, there exists an optimal modal interaction term M_{ij}^* such that for any visual feature v_i and text feature t_j:*

$$\|W_q^l v_i - W_k^l t_j\|_2^2 \leq \varepsilon + 2M_{ij}^* \quad \forall i, j$$

where ε is a small quantity related to d_k, representing the upper bound of cross-modal feature alignment error.

Proof 5 *Let's derive the alignment guarantee with detailed steps:*
First, express the squared L2 norm:

$$\|W_q^l v_i - W_k^l t_j\|_2^2 = \|W_q^l v_i\|_2^2 + \|W_k^l t_j\|_2^2 - 2(W_q^l v_i)^T (W_k^l t_j)$$

The visual feature norm can be expanded as:

$$\|W_q^l v_i\|_2^2 = \sum_{p=1}^{d_k} (w_{qp}^l v_{ip})^2$$

The text feature norm can be expanded as:

$$\|W_k^l t_j\|_2^2 = \sum_{p=1}^{d_k} (w_{kp}^l t_{jp})^2$$

The cross-attention score can be rewritten:

$$\frac{(W_q^l v_i)^T (W_k^l t_j)}{\sqrt{d_k}} = -\frac{1}{2}\|W_q^l v_i - W_k^l t_j\|_2^2 + \frac{1}{2}(\|W_q^l v_i\|_2^2 + \|W_k^l t_j\|_2^2)$$

By the convergence theorem:

$$\frac{(W_q^l v_i)^T (W_k^l t_j)}{\sqrt{d_k}} + M_{ij} \leq \frac{(W_q^l v_i)^T (W_k^l t_{j^*})}{\sqrt{d_k}} + M_{ij^*} + \frac{\varepsilon}{2}$$

Define the optimal modal interaction term:

$$M_{ij}^* = \frac{1}{2} \min_{i,j} \|W_q^l v_i - W_k^l t_j\|_2^2$$

The optimization problem becomes:

$$\min_{M_{ij}} \mathbb{E}[\|W_q^l v_i - W_k^l t_j\|_2^2] \quad \textit{subject to} \quad Var[M_{ij}] = \mathcal{O}(1/d_k)$$

The solution satisfies:

$$\mathbb{E}[M_{ij}^*] = -\frac{1}{2}\mathbb{E}[\|W_q^l v_i - W_k^l t_j\|_2^2]$$

The variance constraint ensures:

$$Var[M_{ij}^*] \leq \frac{C}{d_k}$$

Where C is a constant independent of d_k.
The alignment error can be bounded:

$$\|W_q^l v_i - W_k^l t_j\|_2^2 \leq \|W_q^l v_i - W_k^l t_{j^*}\|_2^2 + \varepsilon + 2M_{ij}^*$$

By definition of j^*:

$$\|W_q^l v_i - W_k^l t_j\|_2^2 \leq \varepsilon + 2M_{ij}^*$$

The error term ε can be bounded:

$$\varepsilon \leq \frac{C_1}{\sqrt{d_k}} + \frac{C_2}{d_k} \log N$$

Where C_1 and C_2 are constants, and N is the sequence length.
